# P-1228. Predictive Performance of Population Pharmacokinetic (Pop PK) Models in InsightRX® for Model-Informed Precision Dosing (MIPD) for Cefepime

**DOI:** 10.1093/ofid/ofae631.1410

**Published:** 2025-01-29

**Authors:** Christina Konig, Joseph L Kuti, Andrew J Fratoni

**Affiliations:** Hartford Hospital, Hartford, Connecticut; Hartford Hospital, Hartford, Connecticut; Hartford Hospital, Hartford, Connecticut

## Abstract

**Background:**

MIPD is a promising Bayesian tool to ensure therapeutic antimicrobial concentrations, with multiple models often available. Model selection and sampling strategy might generate different estimates for different populations. Herein, we assess performance of two models in InsightRX® to predict cefepime PK and exposure through differing sampling approaches.

**Figure:**
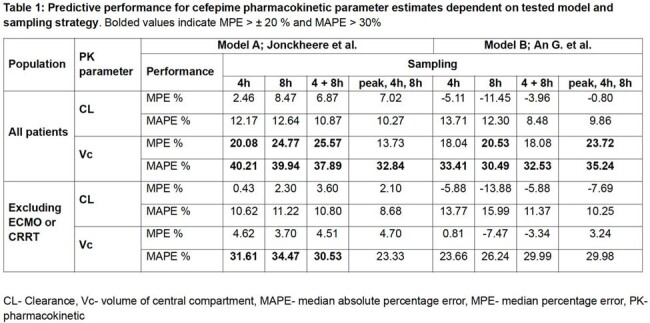

**Methods:**

Historic cefepime PK data were sourced from prospective-observational studies that used robust sampling to develop pop PK models in patients with diabetic foot infection (n=5), VAP (n=15), and supported on ECMO (n=6, 2 on CRRT) or CRRT only (n=4). Historic individual Bayesian estimates served as the reference standard. Two pop PK models (A; B) in InsightRX® were evaluated using four sampling scenarios 1) trough, 2) midpoint, 3) trough and midpoint and 4) peak, midpoint and trough. The median percentage error (MPE) and median absolute percentage error (MAPE) with acceptable defined as < ±20 and < 30%, respectively, were calculated as measures of bias and imprecision for clearance (CL) and volume of central compartment (V_c_). Predicted categorical achievement of ≥ 70% *f*T > 8 mg/L was compared.

**Results:**

Imprecision and bias for CL and V_c_ showed variability dependent on model and sampling strategy (Table 1). MAPE/MPE_CL_ for all models and sampling strategies were acceptable. Both models showed the lowest bias on CL for sampling scenario 4. In the full patient cohort, MAPE/MPE_Vc_ were >30 and > ± 20% for most tested scenarios. When excluding patients with extracorporeal support (i.e. ECMO or CRRT), bias and imprecision for V_c_ improved, with best prediction in model A using sampling scheme 4 and sampling 1 in model B. Using each model and sampling scheme, only 1 patient had discordant predicted categorical achievement of ≥ 70% *f*T >8 mg/L.

**Conclusion:**

Among patient populations representative of the two studied PopPK models in InsightRX®, predicted PK parameters and achievement of relevant exposures demonstrated acceptable performance for clinical care. While all sampling scenarios were acceptable in predicting CL, increasing the number of samples up to three generally improved performance. Appropriate model selection for special populations such as ECMO and CRRT remains paramount as V_c_ was poorly predicted among this subgroup.

**Disclosures:**

**Christina Konig, PhD**, Gilead Inc.: Honoraria|Pfizer Inc.: Honoraria|Shionogi Inc.: Honoraria **Joseph L. Kuti, PharmD**, Abbvie: Advisor/Consultant|bioMerieux: Grant/Research Support|Merck: Grant/Research Support|Pfizer: Grant/Research Support|Shionogi Inc: Advisor/Consultant|Shionogi Inc: Grant/Research Support|Shionogi Inc: Honoraria|Venatorx: Grant/Research Support **Andrew J. Fratoni, PharmD**, InsightRX: Grant/Research Support

